# A retrospective study assessing RefluxStop surgery for gastroesophageal reflux disease: Clinical outcomes in 79 patients from Germany

**DOI:** 10.1016/j.sopen.2024.12.003

**Published:** 2024-12-18

**Authors:** Thorsten Lehmann, Mantas Šimkus, Christoph Oehler

**Affiliations:** Klinikum Friedrichshafen GmbH, Department of Visceral Surgery, Röntgenstraße 2, 88048 Friedrichshafen, Germany

**Keywords:** Hiatal hernia, Gastroesophageal reflux disease, GERD, Fundoplication, Antireflux surgery, RefluxStop

## Abstract

**Background:**

This study reports outcomes of the RefluxStop procedure treating gastroesophageal reflux disease (GERD) in clinical practice at a high-volume regional hospital in Germany.

**Methods:**

A retrospective analysis was conducted on 79 patients with chronic GERD that underwent the RefluxStop procedure, comprising high mediastinal dissection, loose cruroplasty, esophagogastroplication between vagal trunks, and fundus invagination of the RefluxStop implant. The primary outcome was GERD Health-Related Quality-of-Life (GERD-HRQL) score and improvement from baseline. Secondary outcomes included proton pump inhibitor (PPI) use and intra- and postoperative complications, including dysphagia, esophageal dilatation, and reoperation.

**Results:**

Baseline characteristics (*n* = 79) included large hiatal hernia >3 cm (32.4 %) and previous antireflux surgery (20.3 %). At mean (SD) follow-up of 11 (4.4) months ranging from 4 to 19 months, the median (IQR) and mean (SD) improvements in GERD-HRQL score were 100 % (90.2–100 %) and 92.4 % (13.9 %) from baseline, respectively. Significant reduction in PPI use was observed from a baseline of 94.9 % to 2.5 % at follow-up. All cases of preoperative dysphagia (7.6 %) completely resolved. New-onset, mild dysphagia occurred in one subject (1.3 %) at final follow-up. One subject (1.3 %) experienced asymptomatic device migration into the stomach, likely due to surgical technique with a much too tight invagination, with subsequent conversion to Toupet fundoplication.

**Conclusion:**

Analysis of this cohort that underwent RefluxStop surgery indicates excellent safety and effectiveness over this short-term follow-up. Significant improvements in quality of life and PPI use were observed in a population where half had either large hiatal hernia >3 cm or reoperation for previously failed antireflux surgery, a demographic with usually much higher complication rates.

## Introduction

Gastroesophageal reflux disease (GERD) presents with troublesome symptoms, such as heartburn and regurgitation, and posits a substantial global burden that afflicts approximately one-fifth of persons in Western cultures [1]. Proton pump inhibitor (PPI)-based medical therapy does not address the underlying pathophysiologic processes of GERD [2] which likely contributes to the significant rate of drug irresponsiveness [3], and the standard-of-care antireflux procedure (i.e., Nissen fundoplication) can be associated with important postoperative sequelae (i.e., dysphagia, bloating, and the inability to belch or vomit) [1] resulting from wrapping the food passageway to support the closure of the lower esophageal sphincter (LES).

The RefluxStop™ (Implantica AG, Switzerland) procedure is a laparoscopic surgical treatment that received CE mark in Europe in 2018. The procedure aims to correct the defunct mechanisms of the antireflux barrier (ARB) by realigning the angle of His, repositioning the LES, and reinstating the gastroesophageal flap valve using limited esophagogastroplication (90–120°) between the vagal trunks [4], with a different mechanism of action not encircling the food passageway as in traditional techniques such as Nissen fundoplication. Furthermore, the RefluxStop device is invaginated in a pouch on the outside of the gastric fundus to provide regional stability, maintaining the LES intraabdominally during dynamic respiratory and alimentary processes. With this, LES contractility is likely unaffected by the pressure gradient (between the thorax and abdomen) that can vary with respiration. Thus, comprehensive correction of normal ARB anatomy and physiology serves to prevent reflux of gastric contents into the esophagus [5,6]. Moreover, the concert of steps in RefluxStop surgery eludes the necessity for esophageal encirclement, postulated to reduce the manifestation of postoperative complications of traditional fundoplication [4].

Such postoperative complications are acknowledged as important shortcomings of traditional laparoscopic fundoplication [7,8]. Dysphagia may be considered as acute, attributable to surgery-induced inflammation and edema, or persistent, a consequence of fundoplication-related stenosis, potentially attributable to a too tight or too long fundoplication, excessive esophagogastric junction angulation or protrusion of prosthetic surgical material where applicable (e.g., mesh), or disorders of esophageal motility preventing peristalsis from sufficiently overcoming the fundoplication [7]. Acute dysphagia can present in around 50 % of patients [7]; persistent dysphagia is less common, affecting some 10–22 % of patients [7,9], but is arguably more problematic. A recently published literature review of 63 randomized controlled trials showed 22.4 % dysphagia after Nissen fundoplication at 1 year [9]. A meta-analysis of >6000 patients reported gas-bloat syndrome occurred in 26–47 % [10] of patients (laparoscopic Toupet fundoplication and laparoscopic Nissen fundoplication [LNF], respectively). It is well-established that postoperative dysphagia remains a relevant and potentially burdensome complication of fundoplication and alternative techniques (e.g., magnetic sphincter augmentation [MSA]) [[11], [12], [13]]. In the management of GERD, certain clinical features can make the condition more difficult to treat, including large hiatal hernia and ineffective esophageal motility. Large hiatal hernia further substantially enhances the risk of reherniation and failure [1,14]. There are reports of favorable clinical outcomes with RefluxStop surgery in patients with ineffective esophageal motility (IEM) (i.e., low rates of postoperative sequelae); the results have been published recently and present a promising treatment alternative [15,16].

RefluxStop surgery is now offered at numerous institutions throughout Europe with the first outcomes of clinical study published in 2020 [4] and has been offered at our institution since 2021. This report presents the real-world clinical outcomes from the first cohort of patients to undergo RefluxStop surgery at our institution in Germany.

## Methods

### Study design

A retrospective chart review that included recent follow-up visits was conducted to analyze clinical outcomes of those who underwent RefluxStop surgery between July 6th, 2021, and November 22nd, 2022. All patients who underwent the procedure at our regional public hospital in Germany, a high-volume center for foregut surgery, with a minimum of 3 months follow-up were included (*n* = 79).

Treatment with RefluxStop was offered as one of the antireflux surgical options to patients who suffered from GERD with ineffective control of symptoms while receiving long-term PPI therapy and to those unable or unwilling to continue lifelong medical therapy. Patients with hiatal hernia larger than 8 cm in axial length (including partial or complete upside-down-stomach), achalasia or hypercontractile motility disorders, known allergy to silicone, and over 75 years of age were considered unsuitable for RefluxStop surgery. Patients were informed regarding the relative novelty and limited long-term published data of this procedure at the time of surgery. All patients provided informed consent to undergo RefluxStop surgery. As this treatment was not a deviation from the standard of care at our institution, an institutional review board was not required.

### Surgical procedure

The general steps of the surgical technique have been described in a previously published study [4]. The esophagus is liberated up to 10 cm in the mediastinum (measured from the hiatal opening) creating a minimum of 4 cm of abdominal esophagus, but up to 5 or 6 cm is preferable depending on individual patient anatomy. To achieve this intraabdominal length, gentle traction on the esophagus into the abdomen may be applied with extensive mediastinal dissection. Care is taken to leave the pleura intact. The hiatus is closed with figure-of-eight sutures placed predominantly on the dorsal aspect of the esophagus. The width of the hiatal opening is calibrated using a 35 French tube placed in the esophagus and requires free space in the hiatal opening for insertion of a surgical peanut. In patients with thin muscular columns of the left or right crura, as well as those with large hiatal hernia over 3 cm in size, a circular long-term resorbable mesh Ø 7.6 cm Phasix (BD) was placed around the esophagus in a keyhole fashion and on the hiatal musculature. Mesh is not a standard component of the RefluxStop procedure per the device instructions for use; rather, its use was employed according to the operating surgeon's clinical judgement and established practice. This occurred in 32 patients (40.5 %) in this cohort.

**Fig. 1:**
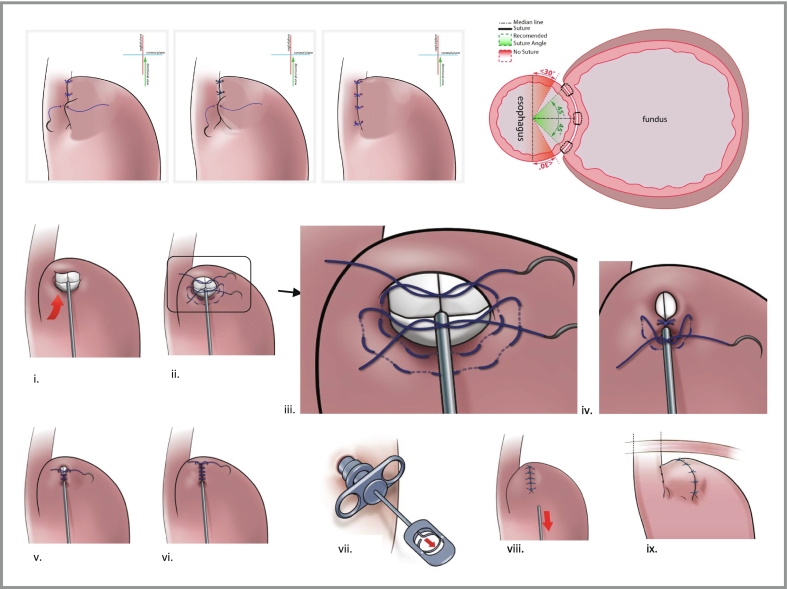
Illustration of portions of RefluxStop surgery. The upper panel shows the limited (minimum 90°) plication performed to approximate the esophagus and gastric fundus. The lower panel shows implantation of the device, via a dedicated deployment tool, in a pocket on the cranial-most aspect of the gastric fundus. *Image provided by the manufacturer of RefluxStop (Implantica, Zug, Switzerland) with permission for use.*

The fundus and esophagus were approximated to result in an overlap around the left aspect of the esophageal wall of at least 90° to 120° (see Fig. 1). A single suture was placed between the two suture rows at the cranial end, close to the diaphragm, to avoid any accordion effect.

The implant was invaginated in a pouch formed with gastric fundus wall (see Fig. 1), close to the esophagus, with the bottom of the implant positioned at least 1 cm above the uppermost border of the LES. Care was taken to ensure complete closure of the pouch with a purse-string suture at the lower end—a crucial step to secure the device position.

### Preoperative assessment

GERD diagnosis was confirmed with standard esophagogastroscopy as a prerequisite for all subjects within 12 months before surgery. A specimen of distal esophagus or cardia was obtained for pathology evaluation in most cases. Esophageal pH measurement was performed only in subjects without endoscopy-proven erosive esophagitis. High-resolution manometry was performed if the indication for antireflux surgery was unclear or a severe motility disorder, including hypercontractile disease of the esophagus, was suspected. A preoperative water-soluble contrast-swallow imaging evaluation was performed if results of other preoperative diagnostics were unclear.

Prior to surgery, assessment of subjects in an outpatient clinic included a complete history of disease and symptoms. A baseline score for symptom severity was determined using the GERD Health-Related Quality of Life (GERD-HRQL) questionnaire (possible score 0–50) [17]. GERD symptoms (i.e., heartburn, regurgitation, bloating, and dysphagia) were categorized as “typical” or “general”.

### Postoperative outcomes

The primary outcome included pre- and postoperative GERD-HRQL scores with calculation of percentage change from baseline to final follow-up. PPI medications were not altered for the purposes of GERD-HRQL scoring (i.e., those patients taking PPIs continued them), however, only two patients took PPIs at follow-up. Secondary outcomes included the incidence of patient-reported symptoms, comparative PPI use since baseline, and the incidence of dysphagia. The incidence of dysphagia was obtained from medical records; subsequently, individual subjects identified as having postoperative dysphagia were assessed using the Dysphagia Numerical Rating Scale (NRS). Intra- and postoperative adverse events (AEs), including reoperation and postoperative esophageal dilatation, were also reported.

### Statistical analysis

Safety and effectiveness outcomes of RefluxStop surgery were analyzed for all subjects. For descriptive analysis, continuous data was summarized by minimum, maximum, median, quartiles (Q25; Q75), mean, and standard deviation (SD); categorical parameters were summarized using absolute counts and percentages. Distribution of continuous data was analyzed by histograms, box plots, and scatter plots. Total GERD-HRQL score change from baseline to follow-up was assessed absolutely and relatively.

Continuous data were tested for normal distribution via the Shapiro-Wilk test. For comparison of baseline and follow-up values, normally distributed data and non-normally distributed data were compared via the Student-t and Mann-Whitney *U* tests, respectively. For quantitative variables, differences between absolute counts were tested using the Pearson's chi-squared test or Fisher's exact test.

Unless stated otherwise, subjects with missing data for an analyzed parameter were excluded from respective analysis with the remaining subjects set to 100 %. Differences were considered significant if the *p* values were below 0.05. Statistical analysis was performed using R version 4.2.27.

## Results

### Sample population

Seventy-nine patients (*n* = 79) underwent RefluxStop surgery with a mean (SD) follow-up of 11 (4.4) months (min-max. 4–19 months). Baseline characteristics are outlined in Table 1. Notably, at baseline subjects had large hiatal hernia >3 cm in axial length (32.9 %) of mean size 5.2 cm (range of 3.5–7 cm with one outlier of 15 cm); previous antireflux surgery (20.3 %); general reflux symptoms (98.7 %) such as heartburn, regurgitation, bloating, dysphagia, and/or other reflux symptoms, of median (IQR) duration 5 (3−10) years (min-max. 6 months-50 years); median (IQR) percentage of total time with esophageal pH <4 of 6.3 % (4.3–14.3 %), of which data was available for 22 subjects (*n* = 22); and median (IQR) DeMeester score of 23.4 (11.8–57.7), in which a pathologic score is considered ≥14.72.

**Table 1:** Patient demographics and baseline characteristics.

Characteristic	
Sex, male, n (%)	47 (59.5 %)
Age, mean (SD), years	49.4 (14)
BMI, mean (SD)	26.8 (4.8)
Duration of symptoms, median (IQR), years	5 (3–10)
Hiatus hernia >3 cm, n (%)	24 (32.4 %)
Previous surgery, n (%)	16 (20.3 %)
Esophagitis, n (%)	
*Yes*	37 (46.8 %)
*No*	27 (34.2 %)
*NA*	15 (19 %)
Barrett's esophagus	
*Yes*	13 (16.5 %)
*With dysplasia*	2 (15.4 % of Barrett's subgroup)
*Without dysplasia*	10 (76.9 % of Barrett's subgroup)
*Dysplasia information NA*	1 (7.7 % of Barrett's subgroup)
*No*	48 (60.8 %)
*NA*	18 (22.8 %)

Abbreviations: BMI, body mass index; NA, not available.

### Primary outcome: GERD-HRQL score

At a mean follow-up of 11 (4.4) months, the median (IQR) total GERD-HRQL score decreased (i.e., improved) by 100 % (90.2–100 %) from a baseline of 21 (18–25) to 0 (0–2) after surgery (*p* < 0.05), as depicted in Fig. 2A. Similarly, the mean (SD) total GERD-HRQL score decreased by 92.4 % (13.9 %) from a baseline of 22.3 (6.0) to 1.7 (3.0) after surgery. Both median and mean reductions were analyzed as the baseline GERD-HRQL scores did not represent a normal distribution curve. The incidence of patient-reported general reflux symptoms decreased from 98.7 % (*n* = 78/79) at baseline to 2.5 % (*n* = 2/79) at follow-up, as shown in Fig. 2B. More specifically, regurgitation was reported in nine (11.4 %) subjects at baseline with complete resolution after surgery; however, de novo regurgitation occurred in two (2.5 %) subjects. Despite these new-onset cases of regurgitation, one of these subjects experienced significant improvement in total GERD-HRQL score (i.e., baseline of 22 decreased to 4 at follow-up).

**Fig. 2A:**
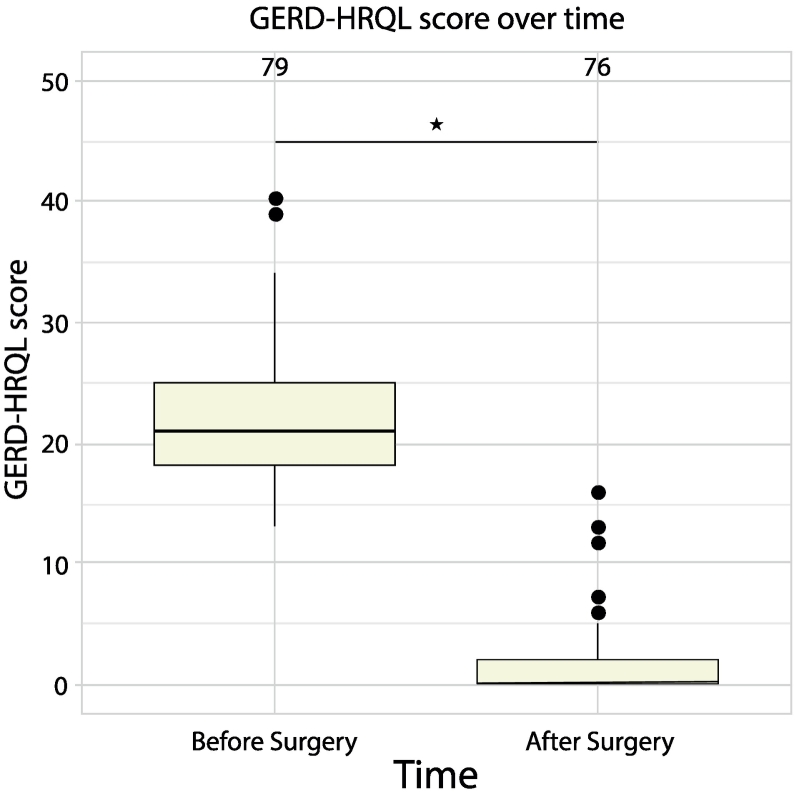
Median GERD-HRQL total score over time. Numbers over each time indicate absolute subject numbers. * *p* < 0.05.

**Fig. 2B:**
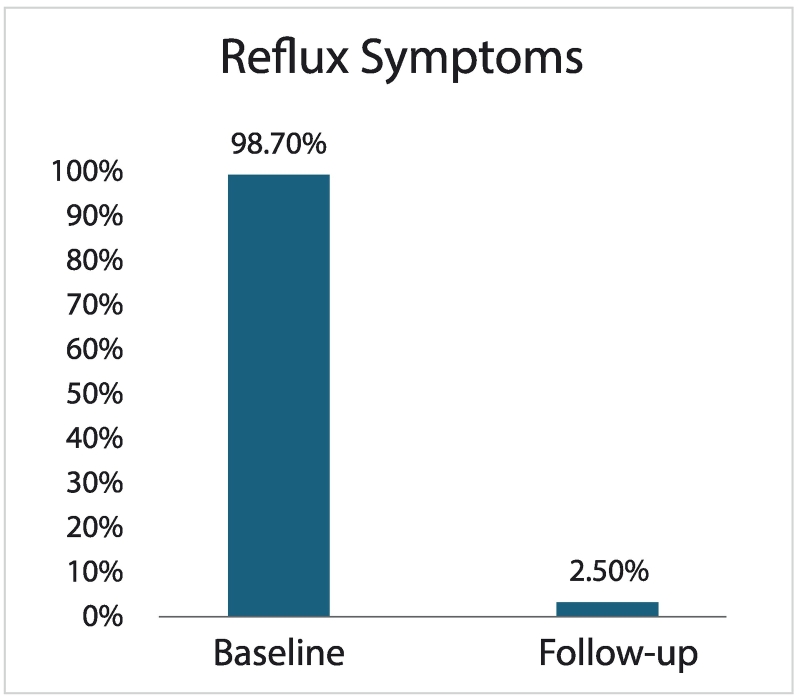
Typical or “general” symptoms of reflux disease (i.e., reported as heartburn, regurgitation, bloating, dysphagia, and/or other reflux problems) at baseline and follow-up.

In addition to the overall patient cohort, we also performed further assessment of the subgroup of patients having large hiatal hernia >3 cm in size, present in 26 patients (32.9 %). We found consistently similar improvement in total GERD-HRQL score change from baseline and in PPI use at follow-up. The GERD-HRQL score was reduced from 23.3 to 1.8 at follow-up at an average of 1 year after surgery. The improvement in GERD-HRQL score from baseline was a mean of 92.3 %. No patient used PPIs at follow-up. Overall, these results are nearly identical with the full group of 79 patients.

### PPI use

Regular daily PPI intake decreased from 96 % (*n* = 72/75) at baseline, with a median (IQR) duration of use for 3 (1–3) years (min-max. 6 months-30 years), to 2.5 % (*n* = 2/79) at follow-up, as depicted in Fig. 2C. However, it is unclear whether PPI use in these two patients was for management of GERD symptomatology; although not performed, 24-h pH monitoring would be of benefit to elucidate this clinical context.

**Fig. 2C:**
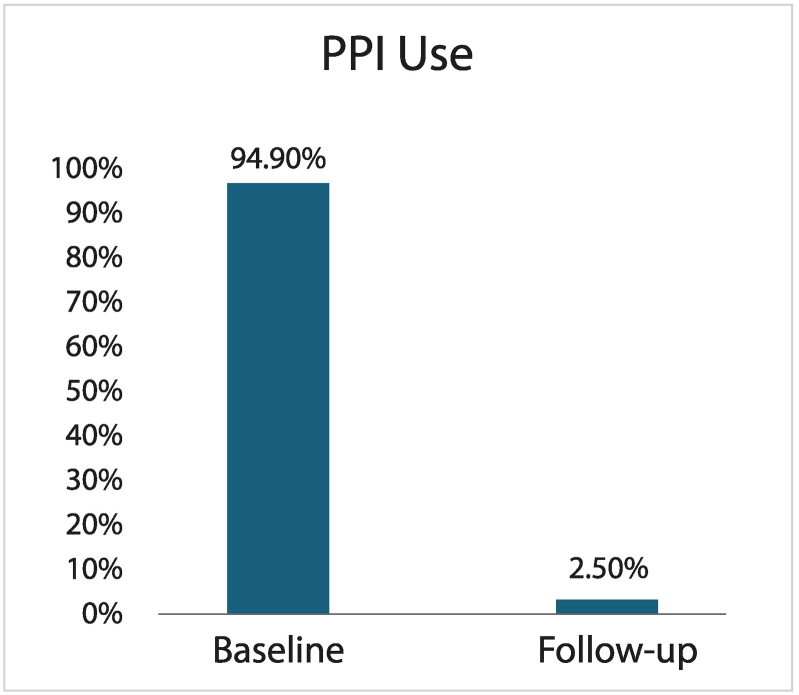
Regular daily PPI use at baseline and at follow-up. GERD-HRQL, Gastroesophageal Reflux Disease Health-Related Quality of Life; PPI, proton pump inhibitor.

### Adverse events

No notable perioperative complications occurred. The most common perioperative AEs included surgical emphysema (i.e., mediastinal and subcutaneous). Altogether, 10 subjects had emphysema that resolved spontaneously 2 to 3 h postoperatively. Two subjects had pleural breach and one subject experienced unusual pain with anesthetic recovery longer than 3 h of duration.

Dysphagia was present in 7.6 % of subjects at baseline with complete resolution after surgery. One subject developed new-onset dysphagia (1.3 %) with a subsequent Dysphagia NRS questionnaire (possible score 0–10) score of 4. No esophageal dilatation or active intervention was required.

One (*n* = 1) patient underwent reoperation at 9 months for conversion to Toupet fundoplication due to early asymptomatic penetration of the device into the gastric cavity. Further review of the case delineated that early penetration was likely due to excessively tight suturing of the invagination pouch. One (n = 1) death due to comorbid cardiac disease occurred during follow-up that was unrelated to the procedure.

## Discussion

The clinical outcomes reported in this study at a mean (SD) follow-up of 11 (4.4) months indicate that RefluxStop surgery is a safe and effective treatment option for patients with chronic GERD.

### Regarding effectiveness

Effectiveness was demonstrated by statistically significant improvements in health-related quality of life, wherein, the mean total GERD-HRQL score (i.e., 10 questions with a maximum 50 points) was 1.7 at follow-up with a mean improvement of 92.4 % and median (IQR) improvement of 100 % (90.2–100 %). Our results add to a growing clinical evidence base indicating that RefluxStop surgery provides favorable outcomes in patients with reflux disease, including subgroups that are arduously managed (i.e., large hiatal hernia, reoperation for other failed antireflux surgeries, and/or comorbid IEM) [15,16,[18], [19], [20]]; see section *Indirect comparison to the RefluxStop CE study*. The large hernia subgroup (32.9 %) with >3 cm hernia size, provided nearly identical mean GERD-HRQL scores of 23.3 at baseline reduced to 1.8 at follow-up at a mean of 1 year after surgery. The improvement was 92.3 %.

Furthermore, patient-reported outcomes of general reflux symptoms exhibited substantial reduction with this intervention, which decreased from 98.7 % at baseline to 2.5 % at follow-up (Fig. 2B). This observation closely aligned with the reduction in regular daily PPI use from 96 % at baseline to 2.5 % after surgery. Notably, only two (*n* = 2) subjects continued PPI therapy after surgery but the reason for this was not qualified further with objective 24-h pH testing to delineate whether medication use was in fact reflux-related. In the large hernia subgroup, no patient required PPI therapy at the final follow-up.

PPI consumption is of particular concern, since it has become apparent that long-term PPI use is plagued with serious adverse effects [2]. In a study by Yan Xie from 2019 [21] following over 157,000 US veterans taking PPI medication for a ten-year period, it was shown that over 7000 excess deaths occurred, or nearly 1 in 20 PPI users. When patients are young, a few years of PPI use may be acceptable, but long-term use may involve high risk. General practitioners and gastroenterologists do not often react due to the long-term nature of side effect manifestation. However, RefluxStop posits a surgical procedure with a novel treatment principle that does not encircle the food passageway and thereby as our results indicate considerably limits AEs such as dysphagia and gas-bloating, that effectively treats the cause of acid reflux unlike PPI therapy, which only reduces the acidity of regurgitated gastric fluid [2].

### Indirect comparison to the RefluxStop CE study

Our experience confers valuable insights and is of particular interest when compared to the excellent 1-year results from the prospective RefluxStop CE study [4], providing additional clinical context in a real-world setting. The CE study is used in the US premarket application for RefluxStop and is a rigorously controlled prospective study. In that trial, with a more strictly controlled population (i.e., hiatal hernia <3 cm and no previous antireflux surgery), the median improvement in total GERD-HRQL score was 95.2 %, the reoperation rate was 2 %, and no regular daily PPI use (0 %) was observed at 1-year follow-up [4]. The 4-year results from the same multicenter study [22] demonstrated that the excellent 1-year results are maintained in the long term, with median 90 % improvement in total GERD-HRQL score from baseline and PPI use in only 4.3 % of patients; normalized 24-h pH monitoring results indicated that PPI use was for reasons unrelated to GERD. Furthermore, no cases of device-related AEs or SAEs, device deficiency, device penetration or migration, device explant, or reherniation occurred during the entire study period [22].

### Regarding safety outcomes

Our analysis showed that RefluxStop surgery resulted in favorable safety outcomes and low rates of AEs despite a considerable proportion of patients having large hiatal hernia >3 cm (32.4 %) and previous antireflux surgery (20.3 %), patient groups that are prone to unfavorable outcomes, risk of complications, failure of hernia repair, and dissatisfaction [7,23].

The low frequency (1.3 %) of mild dysphagia at follow-up is particularly notable since it is the most common side effect of standard-of-care procedures, often reaching 30–50 % [7,9]; see *Naïve-indirect comparison to standard of care*. Moreover, all cases of baseline dysphagia completely resolved after surgery with only one new-onset case at follow-up. Technical execution of hiatus closure is the likely contributing factor to dysphagia with the RefluxStop procedure. It is important to leave slightly more space around the esophagus during hiatus repair as compared to Nissen fundoplication, to avoid contribution to dysphagia that otherwise could be avoided. The RefluxStop procedure does not normally result in dysphagia, and in the 4-year CE mark study results, 46 of 47 patients did not present any such case [22].

One uneventful early penetration into the stomach occurred, resulting in later recurrence of reflux. In this case, a later investigation suggested that the pouch was sutured too tightly. Such an event may be correlated to an anatomically small fundus resulting in an overly tight invagination pouch, thereby stifling blood circulation to the stomach wall. The learning curve of this new procedure involves suturing the pouch loosely as per the instructions for use. An excessively tight device invagination may force the implant through the stomach wall into the stomach cavity, and it is imperative to suture the invagination pouch loosely to avoid such circumstances. However, our thus far gathered experience tells us that circumventing this outcome is predominantly an issue of surgical technique, important in providing adequate circumstances for this procedure. In the 4-year results of the CE mark study, no penetration and migration occurred during the entire study period [22].

The technical execution of the procedure is critical, and we perform dissection high-up into the mediastinum to the *venae pulmonalis*. To achieve appropriate esophageal length, the fundus needs to be freely dissected, not only along the greater curvature of the stomach (i.e., dividing approximately three short gastric vessels), but also posteriorly and inferiorly around the angle of His. This achieves a floppy tension-free fundus that allows for 5 cm plication of at least 90° of the esophageal circumference, placed between the vagal trunks. Plication on the fundus should be placed posterior to the sail leading to the short gastric vessels to allow for a more ventrally positioned free fundus that avoids tight invagination of the device. The device is designed in five components assembled in the operation theater using resorbable suture, which contributes to the avoidance of any direct reoperative measures related to penetration and migration; if this rare circumstance occurs, the device segments should disassociate, thereby enabling separate segments to pass through the gastrointestinal tract via natural means.

The remaining AEs were minor and involved 10 of 79 patients experiencing mediastinal or subcutaneous emphysema that resolved spontaneously shortly after surgery. Surgical emphysema of this type may occur with any procedure requiring similar laparoscopic access and is normally harmless. Furthermore, a few subjects experienced pleural breach (*n* = 2) and unusual pain with delayed anesthetic recovery (*n* = 1), which was resolved. One case of mortality from comorbid cardiac disease occurred during the study that was not related to the procedure.

### Naïve-indirect comparison to standard of care

Albeit naïve-indirect comparison with heterogeneity of study designs, some juxtaposition of RefluxStop surgery and traditional antireflux procedures can be made. The current standard-of-care antireflux surgery, LNF, is widely performed and generally taken as the established benchmark. Some conclusions from the available literature are provided below. Nijjar et al., in a multicenter randomized controlled trial from Australia and New Zealand, reported rates of postoperative dysphagia for solids and PPI intake of 52 % and 12.5 % with LNF at 1-year follow-up, respectively [24]. A recently published systematic literature review that included 63 studies (with 40 trials) and 2619 patients delineated 1-year rates of daily PPI intake (12.3 %), heartburn and/or epigastric/sternal pain (15.1 %), dysphagia (22.4 %), gas-bloating (30.1 %), and reoperation (6.7 %) after LNF [9]. Furthermore, a systematic review and meta-analysis of LNF and MSA with 273 LNF and 415 MSA patients found continued PPI intake rates of 18.5 % and 18.6 %, respectively [25]. The systematic review and meta-analysis [26] performed and used to inform the Society of American Gastrointestinal and Endoscopic Surgeons (SAGES) surgical guidelines [27] discovered that 28 % of patients undergoing surgical management continued PPI therapy at follow-up, though this was over the long-term (i.e., >5 years).

For context, it must be borne in mind that outcomes may be influenced by multiple factors including, but not limited to, surgical technique, surgeon's experience, and patient selection. Furthermore, differences in reporting criteria, for example absence/presence of symptoms versus grading/quantification and qualification of symptoms, may make some symptoms appear more or less common.

Although only indirect comparison is possible, it is evident that larger meta-analysis provides a better platform for comparison and the difference in outcomes between the standard of care and RefluxStop studies is substantial.

### Strengths and limitations

A notable strength of this study is inclusion of real-world patient demographics that draws from clinical settings outside of a controlled trial environment. Furthermore, the size of this cohort (*n* = 79) is somewhat larger relative to existing published evidence on the RefluxStop procedure [4,15,22,28,29]. All surgeries were performed by the same surgical team: a single lead surgeon (with experience of performing >500 foregut procedures) or an experienced surgeon (>100 foregut procedures) under supervision.

A weakness of this study is the limited follow-up time. Given the novelty of this procedure, some degree of learning curve may also have influenced outcomes, especially in earlier cases. Data were collected retrospectively with the limitations inherent to this. Additional information on pre- and postoperative esophageal pH would also be valuable, but as treatment was unchanged from standard of care at this institution, this was only performed postoperatively in symptomatic patients to determine need for additional treatment. Esophageal pH testing would have provided clinical value in cases of continued PPI intake.

## Conclusion

In this study, RefluxStop surgery showed short-term safety and effectiveness in the management of GERD despite over 50 % of the subjects presenting preoperatively with large hiatal hernia and/or for redo surgery following LNF and MSA. This was demonstrated by a significant reduction in reflux symptomatology which decreased from 78 of 79 (98.7 %) subjects at baseline to 2 of 79 (2.5 %) subjects at follow-up, no regular daily PPI use (97.5 %), substantial improvements in health-related quality of life (i.e., median and mean reductions of 100 % and 92.4 %, respectively), and a favorable safety profile with a lack of postoperative dysphagia (98.7 %). Large hernia patients, comprising approximately one-third of the subjects, showed equally favorable results with 92.3 % improvement of GERD-HRQL score from baseline, and no patients required PPI therapy at the mean follow-up of 1 year. Several studies are in the pipeline and our team eagerly awaits these results, including the 5-year outcomes of the CE study.

## CRediT authorship contribution statement

**Thorsten Lehmann:** Writing – review & editing, Writing – original draft, Conceptualization. **Mantas Šimkus:** Writing – review & editing, Investigation. **Christoph Oehler:** Writing – review & editing, Investigation.

## Author contributions

TL: concept, writing—drafting and critical review. MS and CO collected clinical data and performed critical review. All authors approved the final version of the manuscript.

## Ethics approval

Not required due to no changes in standard of care at the institution. All patients provided informed consent to undergo the surgery as per standard clinical practice, including publication of anonymized data for research purposes.

## Funding

No funding was received for this study.

## Declaration of competing interest

The authors declare the following financial interests/personal relationships which may be considered as potential competing interests: Thorsten Lehmann reports a relationship with Implantica Trading AG that includes: consulting or advisory, speaking and lecture fees, and travel reimbursement. Thorsten Lehmann reports a relationship with Bard/BD (Becton Dickinson and Company) that includes: speaking and lecture fees and travel reimbursement. The other authors declare that they have no known competing financial interests or personal relationships that could have appeared to influence the work reported in this paper.
